# Early recognition of pain: improving colic outcomes in horses in Senegal

**DOI:** 10.3389/fpain.2024.1429849

**Published:** 2024-09-05

**Authors:** B. O. F-X. V. Laleye, Mamadou Seye, Ludovica Chiavaccini

**Affiliations:** ^1^ Département Santé Publique et Environnement, Ecole Inter-Etats des Sciences et Médecine Vétérinaires, Dakar, Senegal; ^2^ Sahelvet Clinique et Pharmacie Veterinaire, Thiès, Senegal; ^3^ Department of Comparative, Diagnostic, and Population Medicine, College of Veterinary Medicine, University of Florida, Gainesville, FL, United States

**Keywords:** acute abdomen, abdominal pain, colic management, horse health, low-income countries, referral time, Senegal

## Abstract

**Background:**

Limited knowledge exists on recognition and treatment of equine abdominal pain in low- and middle-income countries. This study aimed at finding indicators for recognizing abdominal pain, evaluating responses to clinical and behavioral changes, and assessing the impact of timely referral on colic outcomes in a suburban region of Senegal. The final goal was to identify factors that may be leveraged to improve the outcome of horses presented for abdominal pain in Senegal.

**Study design:**

Retrospective, observational cohort study.

**Methods:**

Data from 26 foals and 40 adult horses referred for acute abdomen between 2013 and 2014 and the first semester of 2023 were reviewed. Signs of abdominal pain were grouped into behavioral, posture modification and animal interactions with the environment. Time to referral was defined as the time between the recognition of abdominal pain and referral. The association of time to referral and the outcome was calculated for each subpopulation and compared using logistic regression analysis as appropriate.

**Results:**

A significant proportion of owners (47%) and veterinarians (77.8%) relied on behavioral changes to detect abdominal pain in foals. Most owners referred foals within 24 h, while veterinarians referred within 12 h. Mortality in foals exceeded 50% when referral was delayed by 12 h or more. In adult horses, groomers often were the first noticing behavioral changes (79%), and they referred the horse within three hours, whereas owners typically delayed referral for 24 h or longer, leading to increased hospitalization expenses.

**Limitations:**

The study considered a limited cohort in an suburban area of Senegal. Sourcing complete data was challenging. Additionally, accurately assessing owner experience was difficult due to the participant group's heterogeneity. Absence of a reliable system to measure daily horse-owner interaction time and logistical challenges in the abdominal pain symptom alert chain were also limiting factors.

**Conclusions:**

Early detection is critical for positive colic outcomes in both foals and adult horses. Therefore, raising awareness and providing training to horse owners for prompt recognition of symptoms and referral is essential. This proactive approach aims to improve overall outcomes and reduce the financial burden of equine hospitalization in Senegal.

## 1 Introduction

In Senegal, horses play a pivotal role in various aspects of the country's life, influencing sectors like the military, economy, sports, and leisure (1, 2). The equine industry holds significant importance in the daily lives of many Senegalese households today (1). Similar to the rest of the world (3–5), colic colic is considered a leading cause of mortality among foals and adult horses in Senegal. Colic refers to abdominal pain and can result from various disease processes beyond gastrointestinal issues. While many colic cases in horses can be resolved through medical treatment, some require intensive care or surgical intervention. The colic outcome is closely tied to the time of referral, emphasizing the importance of early identification of abdominal pain signs (6–9). Owners, bearing the primary responsibility for identifying symptoms and determining when to seek veterinary assistance, play a crucial role in this process (8, 10). Despite high confidence among horse owners in the UK and US in recognizing abdominal pain, a study revealed a discrepancy between their knowledge and clinical scenario responses, attributed to a lack of understanding and difficulty in recognizing subtle signs of abdominal pain (10). Little is known about the status of equine abdominal pain recognition and treatment in low- and middle-income countries (11), where factors such as limited resources, inadequate training, cultural diversity, and language barriers often result in animals not receiving basic pain treatment. A pragmatic way to change this situation is to look at the country environment in a critical way and identify areas that need prioritization.

This study aimed to explore the criteria considered by horse owners and handlers when assessing signs of abdominal pain in both adult and neonatal horses in Senegal. The objective of the study were: (a) To recognize indicators used by owners or caregivers to recognize abdominal pain; (b) To evaluate their response to changes in clinical and behavioral parameters; (c) To identify the impact of timely referral on colic outcomes in a suburban region of Senegal.

## 2 Materials and methods

This was a retrospective, observational cohort study. Institutional Animal Care and Use Committee approval was not required due to the retrospective nature of the study. The owner consented to the anonymized use of data with the signed consent to treat.

### 2.1 Data collection

The sample included 66 medical records, 40 from adult horses (>5 years old) and 26 from newborn foals (≤4 weeks old) collected from two distinct databases. The dataset comprised the medical records from 24 adult horses and 22 newborn foals referred to the hospital of the National Stud Farm in the Kébemer department, within the Louga region, between 2013 and 2014. At the national stud, all horses showing abdominal pain belonging to the stud herds are supported financially. For horses with colic that are boarding or hospitalized, a flat fee is charged to the owner. Additionally, records from 16 adult horses and four newborn foals collected between January and June 2023 were included from the database of the SahelVet ambulatory practice located in Ngaye Mékhé, within the Tivaouane department, in the Thiès region of Senegal. This practice offers standardized treatment bundle for colic encompassing a clinical examination, transrectal palpation, nasogastric catheterization, and administration of sedatives or antispasmodics. If the horse's general condition requires more intensive management, such as parenteral rehydration or multiple additional visits, the owner is charged accordingly. At the National Stud Farm hospital, all information retrieved from admitted veterinary patients are documented daily in a Microsoft Word Document (Microsoft Corporation, Redmond, WA) saved on a local computer. While at the private equine practice, data are recorded daily in an official paper register. For analysis purposes, only cases with complete and accurate records were retained.

### 2.2 Referring personnel

The referring personnel was categorized into three groups: horse owners (regardless of ownership duration, experience, or practiced sport), veterinarians responsible for routine evaluations of newborn foals at the Kébemer National Stud, and horse caregivers (such as professional groomers). In the Kébemer department, all foals are required to undergo evaluation by a veterinarian shortly after birth for registration. This assessment involves providing a detailed description of the foal, conducting a comprehensive clinical examination, and administering a tetanus injection. Additionally, the health of the mare is assessed during this visit. At the time of data collection, three veterinarians employed by the Kébemer National Stud were responsible for initially identifying any births from stallions within the national stud. This examination typically occurred within 12 h of a reported birth, although delayed notifications were possible due to owner negligence or if the foal was born outside the National Stud system. The group of horse caregivers consisted of 16 groomers employed by the Kébemer National Stud between 2012 and 2014. These caregivers were evenly divided into two groups: one responsible for monitoring mares and the other for stallions. The brood groom group attended to the care of both stud-owned mares and those in boarding for follicular monitoring, insemination, and pregnancy diagnosis. The groom group in charge of stallions ensured the well-being of stallions in the stud. The groomers’ professional experience ranged from 2 to 5 years, with no formal professional training in the field; their expertise was primarily acquired on the job.

### 2.3 Assessment and understanding of abdominal pain in the horse

**Figure 1:**
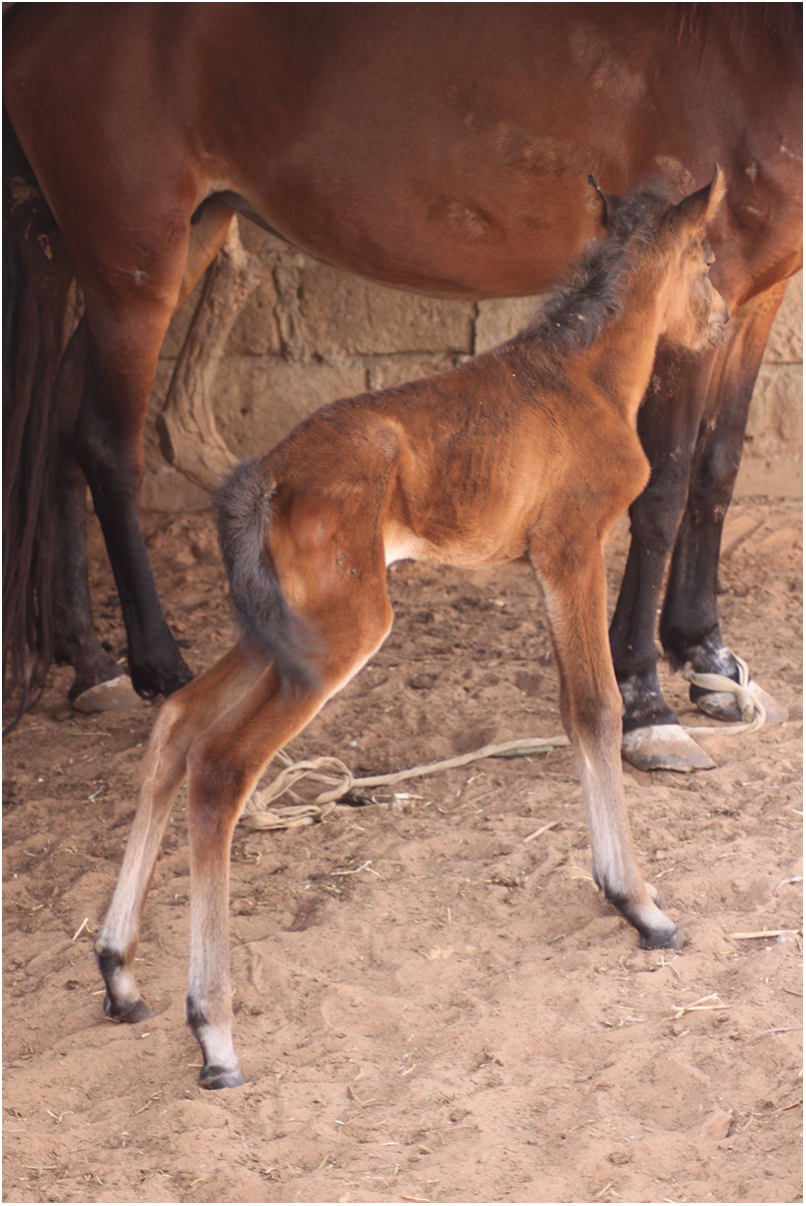
A foal with severe tenesmus, as an example of behavior modification (BM).

**Figure 2:**
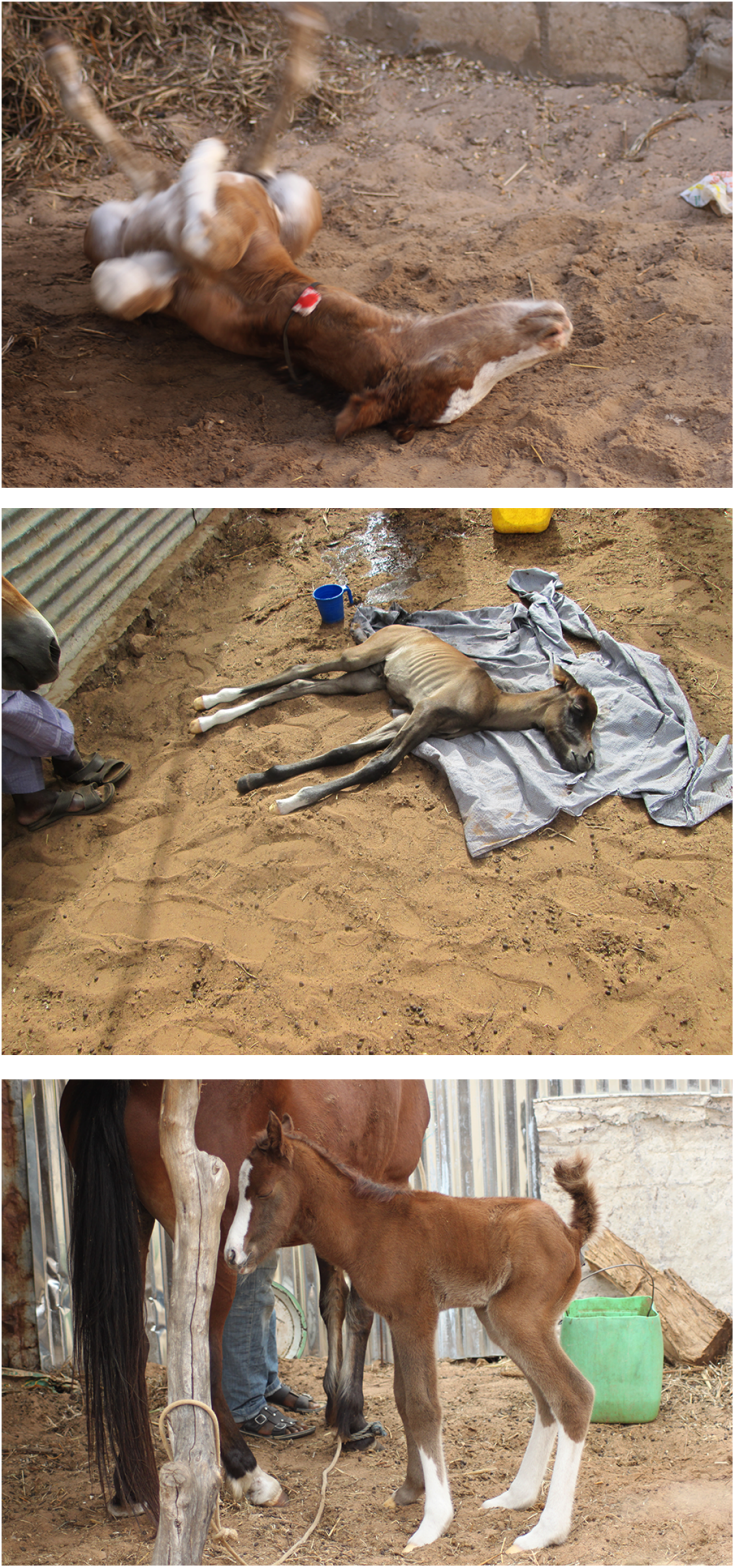
Examples of posture modifications (PM) of foals in colic. **(A)** Lying down and rolling **(B)** lying down motionless **(C)** weight shifting.

Signs of abdominal pain were grouped into three categories for the purpose of the analysis: behavior modification (BM); posture modification (PM) and change in interaction with the environment (IE). In foals BM included: agitation; tenesmus (Figure 1); attempts to defecate and assuming an “abnormal body position” as described by the referring person, and based on the interpretation of the person collecting the history. For adult horses, the BM included: pawing the ground; flank-watching; rolling for an extended period/multiple times; attempt to urinate or defecate, kicking the abdomen and assuming an “abnormal body position”. Change in posture (PM) was defined as weight shifting, fence or box walking, lying down or getting up restlessly or multiple times in both adult and newborn horses (Figure 2). Change in horse IE was defined as an adult or newborn horse appearing abnormally quiet or dull or reported inappetence.

### 2.4 Mortality

Due to the absence of veterinary structures offering equine colic surgery in Senegal, three possible outcomes were identified. The horse condition resolved with medical management, the horse died of spontaneous causes or it was euthanized as a consequence of the condition.

### 2.5 Statistical analysis

All data were entered in a Microsoft Excel worksheet (Microsoft Excel for Mac Version 16.65; Microsoft Corporation, WA, USA). Data elaboration and analysis were conducted with IBM SPSS Statistics and Stata/BE 17.00 (StataLLC, TX, USA). Continuous data were checked for normality using the Shapiro Wilk normality test and graphically with normality quantile plot and histogram distribution. Continuous data were presented as mean ± standard deviation (SD) or median [first and third interquartile (IQR)] if normally or not normally distributed, respectively. Categorical data were presented as proportions and percentage. The association between the referring personnel and the understanding of abdominal pain signs and between time to referral and mortality was compared for each subpopulation using Pearson's chi-square and Fisher's exact tests as appropriate, and using logistic regression. Statistical significance was set at *P* ≤ 0.05.

## 3 Results

The findings are presented separately for newborn foals and adult horses, with each category undergoing individual analysis to underscore pertinent results and trends.

### 3.1 Demographics

All 26 foals evaluated in the study were less than a week old, with 38.4% (10/26) being females. These foals exhibited signs of abdominal discomfort within the first hours after birth, primarily attributed to meconium retention (65.4%; 17/26) and other undiagnosed causes of colic (34,6%; 9/26). Among the 40 reported cases of abdominal pain in adult horses, 52.5% (21/40) were stallions and 47.5% (19/40) were mares. The majority of cases involved indigenous breeds (45%; 18/40), alongside Thoroughbreds (37.5%; 15/40), Arabian Horses (10%; 4/40), and Warmbloods (7.5%; 3/40), reflecting the equine population in the area. The mean ± SD age of adult horses referred for abdominal pain was 11.9 years ± 4.5.

### 3.2 Referring personnel

In cases involving foals, the initial identification of abdominal pain symptoms predominantly stemmed from reports by either owners (65.4%; 17/26) or stud veterinarians (34.6%; 9/26). Owners detected abdominal pain in 11.8% (2/17) of instances within the first 12 h of life, leading to a unanimous diagnosis of meconium retention by attending veterinarians When concerns arose between 12 and 24 h (47%; 8/17), causes of abdominal pain varied, encompassing meconium retention (50%; 4/8), generalized weakness (25%; 2/8), or unspecified gastrointestinal issues (25%; 2/8). In cases where concerns emerged between 24 and 48 h after initial signs (41.2%; 7/17), veterinarians diagnosed meconium retention (57.4%; 4/7), unspecified causes (28.5%; 2/7), and generalized weakness (14.2%; 1/7). Veterinarians identified abdominal pain within the first 12 h of life in 77.8% (7/9) of cases, predominantly addressing meconium retention (85.7%; 6/7) and weak foals (14.3%; 1/7). In the remaining instances (22.2%; 2/9), intervention occurred 24 h after symptom onset, with one case diagnosed as meconium retention and the other as generalized weakness.

In adult horses, the responsibility for identifying symptoms of abdominal pain rested primarily with owners in 52.5% of cases (21/40), while groomers were accountable for 47.5% (19/40) of recognitions. Among owners, 47.6% (10/21) promptly informed the veterinarian within 24 h of symptom recognition. However, a notable proportion (33.4%; 7/21) delayed referral until 48 h after the initial symptom recognition, while a few (9.5%; 2/21) waited even longer, up to 72 h or 120 h (5 days) before seeking veterinary assistance. Owner-initiated therapy was reported in 4/17 (23.5%) abdominal pain cases referred to the veterinarian. In contrast, groomers consistently alerted the referring veterinarian in less than 3 h from the initial recognition of abdominal pain symptoms, ensuring swift intervention.

### 3.3 Assessment and understanding of abdominal pain in the horse

Encompassing both foals and adult horses, owners and veterinarians predominantly relied on BM (only), PM (only) and BM with PM, to identify abdominal pain symptoms (27/47; 57.44%). In contrast, most groomers were prompted by observing changes in the horse's behavior associated with alterations in IE, such as eating habits (16/19; 84.2%), alone or in association with BM (17/19; 89.4%). Only in two instances they relied on PM only. Further details regarding how various demographic groups assessed and responded to signs of abdominal pain within the population are provided in Tables 1, 2.

**Table 1:** Assessment and understanding of colic indicators by referring personnel.

Horses (66)	Referring personnel (66)	Indicators of colic		
		BM *n* (%)	IE *n* (%)	PM *n* (%)
Foals (26)	Owners (17)	8 (47%)	5 (29%)	4 (17%)
	Veterinarians (9)	9 (100%)	–	2 (22%)
Adults (40)	Owners (21)	19 (90.4%)	7 (33%)	2 (9.5%)
	Groomers (19)	17 (89.4%)	16 (84.2%)	2 (10.5%)

BM, behavior modification; IE, interaction with environment; PM, posture modification; n, total number. Some horses were identified has having abdominal pain using two or more combinations of indicators.

**Table 2:** Detection time in case of colic declared by owners, veterinarians and groomers and the relation with mortality.

Horse (66)	Referring personnel		*n* (%)	Mortality *n* (%)	*P*-value
	Personnel (*n*)	Time			
Foals (26)	Owners (17)	<12 h	2 (11.8%)	0	0.44
		12–24 h	8 (47%)	5 (62.5%)	
		24–48 h	7 (41.2%)	3 (42.8%)	
	Veterinarians (9)	<12 h	7 (77.8%)	1 (14.2%)	0.31
		12–24 h	2 (22.2%)	1 (50%)	
Adults (40)	Owners (21)	12–24 h	10 (52.6%)	2 (20%)	0.55
		48 h	8 (36.8%)	0	
		72 h	2 (10.6%)	0	
		>72 h	1 (9.5%)	0	
	Groomers (19)	<12 h	19 (100%)	3 (15.7%)	

n, total number.

### 3.4 Mortality

In cases where foals were referred by owners within 12 h, no mortality was observed. However, when owners referred the foal to the veterinarian within 24 h of initial abdominal pain signs, the mortality rate increased to 57.2% (10/17). If the referral was delayed to 48 h, the mortality rate was 42.8% (3/7). When veterinarians identified a foal suspected of abdominal pain during the registration visit, the mortality rate was 14.2% for interventions within the first 12 h and rose to 50% for those conducted within the first 12–24 h (Table 2).

For adult horses where abdominal pain symptoms were reported by the owner, a mortality rate of 20% (2/10) was observed only if the signs of abdominal pain occurred within 24 h prior to referral. No mortality was observed in other colic cases, regardless of the delay (Table 2). At the Kébemer National Stud, the mortality rate observed when the groomer notified of abdominal pain (always within less than 3 h) was 15.7%. There was no association of mortality with referring personnel, time to referral, or their interaction (*p* = 0.2).

Regarding adult horse abdominal pain cases, the final treatment expenses increased by 33.4% if the horse was referred within 48 h, 66.7% if referred within 72 h, and 80% if referred 120 h or later. No invoice was reported for foals, so a comparison was not possible.

## 4 Discussion

Foals, renowned for their fragility in their early hours, demand careful attention from owners (3). However, owners often struggled to detect the initial signs of abdominal pain in foals, resulting in variable response times. Unlike veterinarians, who swiftly identified abdominal pain based on behavioral changes, owners’ detection capabilities were not as rapid, leading to delayed responses. This observation aligns with findings from a study conducted in Brazil, which aimed to assess horse owners’ experience, recognition, and attitudes towards equine abdominal pain in Rio Grande do Norte (11)*.* Horse owners encountered challenges in identifying and interpreting early abdominal pain warning signs. Our study further suggested that the origin of abdominal pain significantly influenced owners’ detection abilities, as they readily identify overt signs but struggle with insidious conditions with a progressive evolution, such as meconium retention. These conditions would also have the best outcome if properly and promptly addressed (12). On the other hand, situations where owners quickly detected foal distress typically indicated poor prognoses, regardless of management timing.

In cases of adult equine colic, a similar pattern emerged, characterized by disparities in recognized warning signs and highly variable response times. This discrepancy arose from the comparatively lesser attention given to working horses compared to newborn foals (1). Consequently, owners may have overlooked certain early warning signs. Moreover, typically, owners observed their horses only during working hours and meals, with limited contact outside these periods. The location of the equine enclosure may further exacerbate this issue; the farther it is from the owner's home, the less frequently the horse is monitored. However, studies have shown that even when owners spent considerable time with their equine companions, they only provided timely alerts with proper awareness of early colic signs (10). In contrast, professionals like veterinarians and groomers are better trained to assess a horse's condition. In developing countries like Senegal, accessibility to healthcare and limited health education contribute to widespread where owner-initiated medication of horses among the population (13). This habit is also reflected in veterinary medicine, where owners often take the initiative to provide medical care to their animals without direct oversight or intervention from a veterinarian. When owners notice signs of colic, it is not uncommon that they resort to self-initiated therapy, using traditional remedies, before contacting a veterinarian. This practice is prevalent among horse owners, who commonly employ conventional medicinal practices to manage various equine pathologies. In cases of colic, the most frequently used treatment methods include administering decoctions of plants, such as Prosopis Juliflora, through the nostrils or the oral cavity. Some owners opt for alcohol-based solutions, such as beer or wine, using the same routes of administration. In those instances, owners typically seek veterinary assistance only when their self-initiated therapy efforts prove ineffective. Our study found that owner-initiated therapy was reported in 23.5% of colic cases referred to veterinarians. However, this figure may need reassessment, considering that some owners may withhold information about self-administered treatments from the veterinarian. Suspicion may arise during examination upon observing colored (yellow or green) single or bilateral nasal discharge, indicative of nasopharyngeal administration. Respiratory issues, often stemming from aspiration due to improper use of the nasopharyngeal route, are the most common complications in such cases. In all the cases reported in this study, Prosopis Juliflora decoctions were administered orally.

When examining the implications of delayed alerts, it is suggested that in the case of foals, tardy notifications may correlate with reduced survival probabilities, although we were unable to establish a statistically significant association with the small number of subjects. Extensive research underscores the pivotal role of timely referral for newborn foals (14). Conversely, late referrals in cases of adult horse colic did not exhibit a corresponding increase in mortality. Even if the referring personnel were professional and the referral was made promptly, a horse with a surgical reason for colic could still succumb to the condition. Conversely, if the referring personnel were owners and delayed the referral, a horse with a medical reason for abdominal pain could still survive. Therefore, mortality alone did not provide a comprehensive assessment of abdominal pain management in our study. Other factors, such as the nature and severity of the colic and the timeliness and appropriateness of interventions, had to be considered to evaluate the effectiveness of abdominal pain management practices fully. This disparity could also be attributed to the prevalent etiologies of abdominal pain in Senegal, primarily stemming from food impactions or stasis in the large intestine, a consequence of suboptimal feeding practices and inadequate deworming protocols. The survival rates for such colic surpass those observed for colic affecting the small intestine (15). However, it is imperative to acknowledge that delayed notifications necessitate a heightened allocation of resources for horse management. Severe climatic conditions exacerbate dehydration rapidly, necessitating fluid therapy and adjunctive therapeutic measures, thereby magnifying the financial burden associated with medical management. Analogous to observations in foal abdominal pain, owners often overlooked signs indicative of benign conditions, allowing them to deteriorate while demonstrating prompt responses to scenarios associated with lower survival probabilities. Despite these limitations, the overall case fatality rate for adult horses in our study was comparable to that reported in wealthier nations like Canada and the United States, known for their high standard of veterinary medicine, advanced infrastructure, and well-established institutions (15, 16).

### 4.1 Limitations

The study encountered several limitations. Firstly, the challenge of sourcing cases with complete data significantly hindered the research process. Despite a large pool of potential cases, less than 10% of records yielded usable information, highlighting the need for improved medical record keeping. Additionally, accurately gauging the level of owner experience proved challenging due to the heterogeneous nature of the participant group. Another limitation stemmed from the absence of a reliable system to measure the time owners spent with their horses daily, which has potential implications for horse welfare and health. Moreover, the alert chain for abdominal pain symptoms posed a logistical challenge, often involving multiple individuals before reaching the owner, potentially delaying timely intervention. Lastly, the financial implications of veterinary interventions presented a barrier to prompt alertness from owners, contrasting with the swift actions of groomers who prioritized horse well-being over economic concerns.

However, despite these limitations, the study provides an overview of factors that can be leveraged to improve the welfare of horses in Senegal. With its population of 17 million inhabitants and a herd of 1 million equines, including 462,000 donkeys (1), Senegal has fewer than 200 private veterinarians spread across its territory spanning 196,712 km^2^. This notably low rate of veterinary coverage poses challenges in managing equine colic, particularly in rural areas and within the study area. Raising awareness and educating owners is crucial, as abdominal pain management in horses is directly linked to its early detection, a responsibility typically shouldered by owners. This imperative for primary horse education extends to encompassing correct horse handling, feeding, grooming, and pain assessment. Collaboration with local community figures, private veterinarians, and state structures such as the Kébémer Stud Farm is imperative. Various mediums such as local community radio, social media platforms, and direct interaction between professionals and horse owners offer avenues for effective communication and awareness-raising. Recognizing that awareness and communication are as pivotal as increasing the number of private veterinarians and their responsiveness is essential for enhancing equine welfare in Senegal.

## 5 Conclusion

The findings underscore the insufficient and disparate level of knowledge among owners regarding detecting abdominal pain signs in both foals and adult horses, directly impacting mortality rates for foals and financial costs for adult abdominal pain cases. Unlike professionals, owners frequently failed to identify the early, subtle signs of colic with a potentially favorable prognosis in foals and adults, often referring the horse only when the animal's condition had deteriorated. Enhancing equine well-being in Senegal requires sensitizing and training owners to recognize early signs for prompt intervention. Implementing mechanisms tailored to the country's realities, with active engagement from veterinarians and stakeholders in the equine sector, would be crucial steps toward achieving this goal.

## Data Availability

The original contributions presented in the study are included in the article/Supplementary Material, further inquiries can be directed to the corresponding author.
